# Mechanisms of transcriptional regulation of CCR5, which is the co-receptor for R5-tropic HIV and is also involved in other disease processes

**DOI:** 10.1042/BST20253092

**Published:** 2026-01-28

**Authors:** Lingyun Wang, Sharon Veron Akisa, Richard Sutton

**Affiliations:** 1 Section of Infectious Diseases, Department of Internal Medicine, Yale School of Medicine, New Haven, CT, U.S.A.

**Keywords:** CCR5, epigenetics, HIV, signaling pathways, transcription factors

## Abstract

C–C chemokine receptor type 5 (CCR5) is the R5-tropic human immunodeficiency virus type 1 or HIV co-receptor. Lower CCR5 levels can reduce T cell and macrophage susceptibility and suppress HIV infection. Moreover, CCR5Δ32 homozygous stem cell transplantation is central to HIV cure. Other studies have shown that CCR5 plays a vital role in cancer development and cell migration, and it was considered a potential therapeutic target for several types of malignancy. In addition to HIV and cancer, CCR5 also participates in immune response and plays a role in graft-versus-host disease in bone marrow transplant patients. It is also associated with other diseases, such as Parkinson’s disease and rheumatoid arthritis. Thus, investigating its regulatory mechanisms is critically important for understanding the progress and therapeutics of other illnesses. Transcriptional regulation of genes is a complex process that controls when, where, and how much the RNA transcript is produced. In this minireview, we discuss epigenetic regulatory mechanisms, such as DNA methylation and histone modification, transcription factors, and signal transduction pathways, involved in the regulation of CCR5 transcripts.

## Introduction

Transcriptional regulation of genes is a complex process that controls when, where, and how much RNA is produced. Several mechanisms are involved in regulating RNA levels, including chromatin modifications, DNA sequences of the promoter/enhancer, expression of tissue- or cell-type specific transcription factors, signal transduction molecules, microRNAs (miRNAs), and so forth. Each of these mechanisms can interact in complex ways to modulate gene expression, ensuring that the RNA is expressed at the appropriate time, in the correct cell types, at the proper level, responsive to internal and external stimuli, maintaining homeostasis, and differentiation into various cell types.

**Figure 1:**
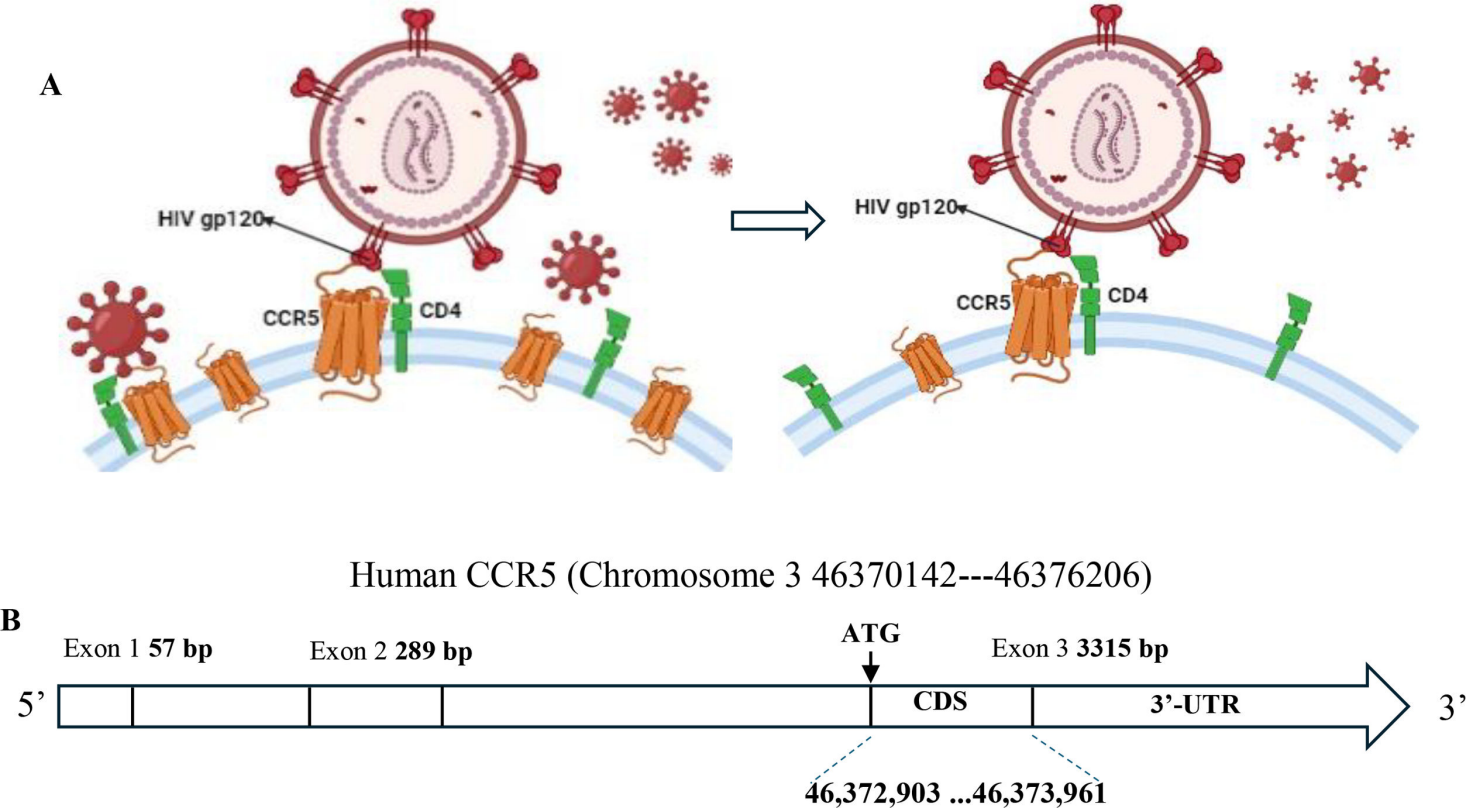
(A) Schematic of CCR5 as R5-tropic HIV co-receptor. Left represents normal CCR5 expression on the cell surface; right shows lower cell surface CCR5 expression. GP120 is a trimer on the surface of HIV, shown in magenta; CCR5 is in orange, and CD4 is green. The CCR5 that binds to HIV is shown larger for illustrative purposes only. (B) Human CCR5 exonic gene structure. The numbers shown at the top are from hg38 of the genomic sequence of the unspliced RNA of CCR5, and in bold at the bottom is the coding sequence or CDS of CCR5 on human chromosome 3 p. ATG is start codon; 3’-UTR is 3 prime untranslated region.

Human C-C chemokine receptor type 5 (CCR5) is located on the short arm of chromosome 3 at position 21. It is expressed on the surface of primarily immune cells and is involved in immune responses [1]. CCR5 is a 7-transmembrane, G protein-coupled receptor for ligands CCL3 [macrophage inflammatory protein (MIP)-1α], CCL4 (MIP-1β), CCL5 (RANTES), and CCL3L1 [2-4]. It also acts as a co-receptor for R5-tropic human immunodeficiency virus type 1 (HIV-1), which causes acquired immunodeficiency syndrome (AIDS). In 2022, the World Health Organization (WHO) reported that there are roughly 40 million people living with HIV in the world, and 1.3 million people were newly infected by the virus [5]. HIV thus remains a global epidemic. Previous research found that lower CCR5 expression on CD4+ T cells decreased T cell susceptibility and reduced virus spread, as CCR5 is the primary co-receptor of R5-tropic HIV entry [6,7]. Moreover, HIV elite controllers, who live with HIV but maintain relatively normal peripheral CD4+ T cell counts and whose HIV viral loads remain at undetectable levels in the absence of antiretroviral therapy (ART) for a prolonged period of time, have lower CCR5 expression levels [8]. A schematic of CCR5 as a co-receptor for HIV is illustrated in Figure 1A. Five patients have been cured of HIV after receipt of homozygous CCR5 Δ32 bone marrow or stem cell transplants [9]. Consequently, it is essential to understand the transcriptional regulation of CCR5 more fully to achieve a cure for individuals who are infected with R5-tropic HIV.

CCR5 is also expressed on certain tumor cells and is involved in malignant cell proliferation, invasion, migration, and survival, and it contributes to the maintenance of the tumor immunosuppressive microenvironment [10]. Increased CCR5 expression in triple-negative breast cancer is associated with better survival of patients with an associated TP53 mutation [11]. Up-regulation of CCR5 in various types of cancer is associated with poor prognosis and may lead to various forms of malignancies, including breast, colorectal, lung, and pancreatic cancer [12-16]. Down-regulation of the CCR5 gene in human prostate cancer cells can suppress cell proliferation and induce cell apoptosis [17], and decreased CCR5 expression also suppresses cell proliferation and cell invasion in cervical cancer cells [18]. Investigators Berger and Hemmatazad summarized the impact of CCR5 and the possibility of it being a therapeutic target and concluded that CCR5 antagonists are a promising option to expand the anticancer therapeutic approach [10]. In summary, CCR5 expressions are central to certain types of cancer progression, and targeting CCR5 may be a useful target for therapy against particular malignancies.

CCR5 also participates in the development of acute graft‐versus‐host disease (GVHD). CCR5 down-regulation can reduce the risk of GVHD after allogeneic hematopoietic stem cell transplantation (allo-HSCT) [19]. Maraviroc, which is a molecular antagonist of CCR5 and Federal Drug Agency (FDA)-approved for treatment of patients with R5-tropic HIV, can effectively block CCR5 and results in decreased peripheral T-cell activation and a low incidence of visceral GVHD [20]. Cenicriviroc, which is a dual antagonist for the chemokine receptors CCR2 and CCR5, significantly reduced the morbidity of GVHD-associated skin and intestine injury through inhibition of CCR5 expression in a rat liver transplantation model [21]. Blockade of CCR5 can efficiently lessen the development of acute GVHD in murine allo-HSCT model [22]. These studies indicate that CCR5 plays an important role in the development of acute GVHD.

In addition to HIV and certain kinds of cancers, CCR5 is associated with other illnesses. CCR5 is involved in many vascular diseases; individuals who are heterozygous for CCR5Δ32 had a higher risk for cardiovascular diseases compared with those with the wildtype CCR5 allele [23]. It was also reported that CCR5-positive T cells were observed in and around pulmonary venous occlusive lesions [24]. In mouse models, investigators observed that CCR5 is involved in aldosterone-induced hypertension, vascular dysfunction, and renal damage [25]. The expression of CCR5 and its ligands was significantly increased in infants with bronchopulmonary dysplasia (BPD) [26]. Its expression was critical in the development of BPD, and it may become a novel therapeutic target for that illness [26]. CCR5 was also shown to be involved in the progression of lung fibrosis on interstitial macrophages (IMs) in a steroid-resistant murine model of severe asthma in which subepithelial fibrosis was more marked and CCR5 expression was significantly higher [14]. Further study showed that CCR5 on IMs affects the production of transforming growth factor-β (TGF-β) in the lungs and, in turn, influences the development of subepithelial fibrosis in severe asthma [14]. CCR5 also plays a role in cerebral ischemia, as well as in both Alzheimer’s and Parkinson’s diseases [27,28]. Moreover, it contributes to autoimmune diseases such as rheumatoid arthritis in Vdelta2 T cells, and elevated expression levels of CCR5 promoted the pathogenesis of rheumatoid arthritis [29]. Its importance has also been recognized in atherosclerosis and multiple sclerosis [30,31]. Hence, understanding CCR5 gene expression is critically important to other diseases besides HIV and certain types of cancer.

Since CCR5 was associated with so many diseases, understanding its regulatory mechanisms and developing therapeutic strategies that target CCR5 is becoming increasingly important. In this minireview, we summarize the factors that are involved in the transcriptional regulation of CCR5, including epigenetics and signaling pathways.

### CCR5 genetic and epigenetic regulation

**Figure 2:**
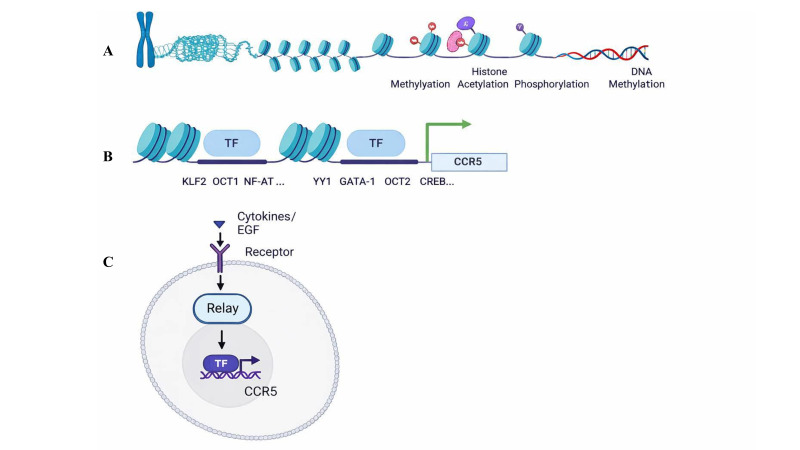
Transcriptional regulation of CCR5. (**A**) Schematic of genetic and epigenetic regulation; (**B**) Transcription factors that could affect CCR5 gene expression; (**C**) Signal transduction involved in CCR5 gene expression. TF denotes transcription factor. Relay denotes intermediate genes that directly or indirectly affect the signaling pathway and result in modulating CCR5 expression.

CCR5 genetic and epigenetic regulation play a critical role in various physiological and pathological processes, including HIV infection [32], cancer [33,34], and other autoimmune diseases. The regulatory mechanisms include histone methylation, acetylation, promoter polymorphisms, and chromatin remodeling, among others (Figure 2A). The regulatory processes have an impact on CCR5 expression levels and affect the progression of diseases [35].

CCR5 variants can significantly influence its expression and function. The most well-known variant is the CCR5Δ32 mutation, which has a 32 base pair deletion in the coding region of CCR5 [36]. Individuals with the CCR5Δ32 homozygous mutation have a nonfunctional CCR5 protein and are highly resistant to R5-tropic HIV infection [37]. Those individuals with CCR5Δ32 heterozygous mutation have reduced CCR5 expression and slower progression to AIDS, which can also confer partial protection against HIV infection [36,38]. Five patients have been cured of HIV after receiving from a donor a homozygous CCR5Δ32 stem cell or bone marrow transplantation. One of the people living with HIV (PLWH) may have been cured after receipt of a heterozygous CCR5Δ32 transplantation, although this person is only 2 years post-transplant. Last year, there was a case report of a man who received an allo-HSCT with wild type CCR5 donor cells to treat an extramedullary myeloid tumor who has had sustained HIV remission for 72 months [39]. This patient was prescribed ruxolitinib to treat chronic GVHD. After stopping ART, his HIV viral load was undetectable, and no virus was amplified in cultures of CD4+ T cells isolated from the patient. After being on ruxolitinib for several months, the cell surface levels of CCR5 on peripheral CD4+ T cells were markedly reduced. This notable development is strong evidence that targeting CCR5 or reducing its level of expression may be the key to curing many more PLWH individuals who carry R5-tropic virus [6,40,41].

Variations in the promoter region of the CCR5 gene may affect CCR5 protein expression. Previous studies have shown that single nucleotide polymorphisms (SNPs) in the CCR5 promoter can result in higher or lower expression of the gene and further affect the susceptibility of host cells to diseases [32,42,43], such as HIV [44], non-small cell lung cancer (NSCLC) [16], and systemic lupus erythematosus [45]. Joshi et al. reported that CCR5 promoter polymorphisms, or SNPs, altered transcription factor binding, leading to differences in CCR5 expression and were associated with progression to AIDS and CD4+ T cell counts in PLWH; thus, these promoter SNPs play a role in regulating HIV disease progression [42]. In 2024, Lu et al. performed an *in-silico* analysis using public data and observed that the lung cancer patients with higher CCR5 expression levels had a greater survival rate than the patients with lower CCR5 expression levels. CCR5 expressions in B cells, CD8+ T cells, and CD4+ T cells are positively correlated with cellular infiltration in lung adenocarcinoma and lung squamous cell cancer. Further study showed that the CCR5 polymorphism haplotype H5: A-G-G-T-G-C increased the risk of NSCLC and also decreased CCR5 promoter transcriptional activity *in vitro* [16]. CCR5 polymorphisms are associated with survival after bone marrow transplantation [46]. Recipients with the CCR5 haplotype H1/H1 demonstrated better disease-free and overall survival [46]. Using polymorphisms in CCR5 to design accurate and targeted therapeutics and new diagnostic strategies to optimize therapy, unfortunately, may be quite difficult at present.

Epigenetic modifications of DNA and histones also contribute to the regulation of CCR5 gene expression levels in related immune cells [35,47,48]. DNA methylation typically suppresses gene expression. The interaction between genetic variations and epigenetic modifications in the regulation of CCR5 expression is a topic of interest. For example, there is evidence supporting the association between genetic variations, such as polymorphisms and CpG site methylations, and CCR5 cis-regulatory regions. This suggests a central epigenetic determinant role of methylation content of CCR5 cis-regions in T-cell CCR5 levels, and possibly HIV-related outcomes [32]. Methylation of the CCR5 promoter region reduced its expression and affected immune responses and T cell susceptibility to HIV. CCR5 expression levels in CD4+ T cells were correlated with CCR5 promoter methylation levels. The higher versus lower promoter region methylation status was associated with lower versus higher CCR5 messenger RNA (mRNA) and cell surface protein expression levels, respectively, and methylation of the promoter region of *ccr5* is able to down-regulate its own expression, potentially reducing the ability of HIV to enter cells [32,49]. Genome-wide DNA methylation arrays were performed on human CD8+ T cells. CCR5 and CCL5 showed consistent methylation patterns in response to CD137 co-stimulation, and both mRNA and protein expression were regulated by differential DNA methylation [50].

Histones are nuclear chromatin proteins that bind to DNA. Histone modifications, including methylation, acetylation, and phosphorylation, can affect chromatin structure and gene expression [47,51]. Regarding CCR5, Wierda et al. showed that CCR5-expressing CD14+ monocytes had higher acetylated histone H3 levels compared with naïve T cells, which do not express CCR5, suggesting that histone acetylation up-regulated CCR5 expression [47]. Basova et al. performed a ChIP-Seq in methamphetamine (Meth)-, Tat-, or dopamine (DA)-stimulated THP1 cells to assess histone modification changes in the CCR5 promoter region, and they demonstrated that Tat alone does not show differences in histone modification levels, which suggests that it does not affect CCR5 transcription directly. Meth, in the presence or absence of Tat, caused a significant increase in the number of cells with H3K27Ac, which is an enhancer modification. DA induced a robust increase in enhancer histone modifications, suggesting that Meth and DA up-regulated CCR5 expression in THP1 cells by promoting epigenetic modifications which enable the transcription factors to bind and initiate transcription [52]. Thus, specific histone modifications can either enhance or suppress CCR5 expression, potentially influencing immune cell function and HIV disease progression.

N6-methyladenosine (m6A) is the most common and abundant epigenetic RNA modification, which controls mRNA metabolism and thus determines cell differentiation, proliferation, and response to stimulation. The m6A methyltransferase-like 3 (METTL3) plays a role in controlling T cell homeostasis and maintaining the suppressive function of regulatory T cells or Tregs. Depletion of METTL3 decreased interleukin-17A (IL-17A) and CCR5 expression via facilitating SOCS3 mRNA stability in Th17 cells [53].

Post-transcriptional regulation also plays a critical role in gene expression. miRNAs are one of the important post-transcriptional regulatory processes. Certain miRNAs are capable of binding to CCR5 mRNA and result in its degradation or inhibition of translation, thus decreasing CCR5 protein levels. Che et al. reported that miRNA107 is a CCR5 regulator and reduced CCR5 expression, which then led to inhibition of cervical cancer proliferation and invasion [18]. In 2022, it was noted that miRNA103 down-regulated CCR5 expression on CD4+ T cells and decreased R5-tropic HIV infection [54]. It has been shown that MiR-455–5p decreased CCR5 expression in prostate cancer cells and then suppressed cellular proliferation and induced cell apoptosis [17]. MiR-153–3p is another reported CCR5 suppressor [55].

The other post-transcriptional regulator is long noncoding RNA (lncRNA), which could bind to target DNA, RNA, or protein and then affect gene expression. In 2019, Kulkarni et al. showed that CCR5AS lncRNA affected CCR5 expression on CD4+ T cells. Inhibition of CCR5AS expression reduced CCR5 expression and enhanced the resistance of CD4+ T cells to HIV infection [56]. They demonstrated the determinant role of some genetic and epigenetic interactions in the functional phenotypes of the chemokine receptor CCR5 in the context of HIV infection and suggested that post-transcriptional regulation of CCR5 expression could occur and be impactful. In addition, chromatin remodeling complexes can alter the structure of chromatin, making it either more accessible or restricted to the transcriptional machinery [57]. These changes can influence the ability of transcription factors to bind to the CCR5 promoter and regulate its expression [8]. Mammalian chromatin remodelers SWI/SNF (SWItch/Sucrose Non-Fermentable) promote the reorganization of nucleosomes along DNA strands [58]. McDonald et al. investigated the role of the canonical BRG1/BRM-associated factor (cBAF) chromatin remodeling complex in antiviral CD8+ T cells during infection [59]. They showed that the cBAF subunit ARID1A was recruited shortly after activation and established new open chromatin regions in the enhancer region. Deficit of ARID1A impaired the opening of thousands of activation-induced enhancers, leading to impaired transcription factor binding and dysregulated cell proliferation/gene expression. Cells in which ARID1A was knocked out expressed high levels of CCR5 [59].

Overall, understanding the genetic and epigenetic regulation of CCR5 raises multiple possibilities for the development of novel therapies against HIV. Even though SNP and CCR5 mutations are difficult to control and specifically target to develop medications, small molecules based on the mechanisms of DNA methylation, histone modifications, and non-coding RNAs may be explored to modulate CCR5 expression for therapeutic purposes.

### CCR5 transcription factors

Transcription factors bind to specific DNA sequence elements, controlling the rate of DNA transcribed into mRNA. The expression of CCR5 is regulated by several transcription factors, which can enhance or suppress its gene expression (Figure 2B). Banerjee et al. showed that cAMP-responsive element (CRE) binding protein 1 (CREB-1) binds to the CCR5 promoter CRE region and increases CCR5 gene transcription in TF-1 cells, which is an immortalized human bone marrow progenitor CD34+, CD4+, CCR5+ cell line [60]. Additionally, Kuipers et al. showed that the cAMP/CREB pathway is involved in the regulation of CCR5 transcription, whereas neither interferon regulatory factor 1 (IRF-1) nor NF-κB, which are thought to be CCR5 transcriptional factors in T lymphocytes, are involved in CCR5 promoter activation [61]. NF-κB, however, activated CCR5 expression in response to pro-inflammatory signals, such as cytokines (e.g. TNF-α and IL-1β) [62]. The NF-κB inhibitor pyrrolidine dithiocarbamate blocked CCR5 expression, whereas RIP3 up-regulated CCR5 expression through NF-κB [26]. Therefore, the mechanisms by which NF-κB regulates CCR5 expression are not clear at present and require further investigation.

Signal transducers and activators of transcription (STATs), such as STAT1, STAT3, and STAT5, are activated by cytokine receptors and play an important role in mediating immune responses. STATs, which eventually become phosphorylated by cytokines such as Interferon γ (IFN-γ), can then enter the nucleus, bind to DNA, and induce CCR5 gene expression in various immune cells [63,64]. Makuta et al. reported that STAT-3 regulates CCR5 expression in immortalized HL60 cells, and the complete stimulation of CCR5 expression required Erk-mediated Ser phosphorylation [65]. Our own results also showed that Janus kinase (JAK) and STATs positively regulate CCR5 expression at both the RNA and protein levels [6]. All these studies indicated that STAT induces CCR5 expression in CD4+ T cells and macrophages. The CHIP-seq database showed that CCR5 is a target gene for multiple STATs, and Makuta et al. reported that there is a STAT binding site in the CCR5 promoter region and that STAT3 enhanced CCR5 transcriptional activity, but they did not verify the functionality of this binding element [65]. In addition, Kanai et al. demonstrated that CCR5 is not a target gene of STAT 5A and 5B in human T cells [66], and no other experiment showed that any STAT protein binds directly to the CCR5 promoter. Additional studies should be performed to more precisely define how CCR5 gene expression is regulated by the STATs.

Forkhead box P3 (FOXP3) is a transcription factor that plays a pivotal role in the function and development of Tregs [67-69]. FoxP3 was also identified as a CCR5 transcription factor and modulated CCR5 expression on Tregs and affected their ability to migrate to sites of inflammation or infection [70]. Recently, Feng et al. reported that inactivated FOXP3 led to reduced CCR5 expression and inhibited HIV infection in immortalized Jurkat T cells [71]. How this regulation occurs, however, is not precisely known.

Runt-related transcription factor 3 (Runx3) is a transcription factor with varied roles in cell differentiation, cancer, and development. Runx3 knockout (KO) blocked T-cell infiltration and down-regulated CCR3 and CCR5 in CD8+ T cells. Runx3 is the key mediator of low-dose decitabine (DAC)-primed anti-PD-1 immunotherapy. DAC can integrate into DNA and bind to DNA methyltransferase, inhibiting its function. In DAC-treated CD8+ T cells, both CCR3 and CCR5 were up-regulated significantly [72]. This is further proof that methylation affects CCR5 expression.

Other studies have revealed that Oct1 and Oct2, NF-AT, YY1, GATA-1/3, AP-1, KLF2, and Sp3 are also CCR5 transcription factors [48]. Nuclear receptors RXR (retinoid X receptor) can interact with other transcription factors to regulate CCR5 gene expression; modulation of CCR5 expression may occur in response to retinoid signaling, although the specific role is less well characterized [73]. Based on these transcription factors, it is likely that new inhibitors will be developed and explored to modulate CCR5 expression and influence diseases in which CCR5 expression plays a role.

### CCR5 regulation-related signaling pathways

The transcriptional regulation of CCR5 involves multiple signaling pathways that influence its expression in response to various stimuli. These pathways include those that respond to cytokines, growth factors (GFs), and other extracellular signals, ultimately affecting the activity of transcription factors that bind to the CCR5 promoter (Figure 2C).

The JAK/STAT signaling pathway is involved in cell growth, cell differentiation and proliferation, and immune response regulation [6]. It also plays an important role in HIV infection and integration [63]. At the same time, the pathway affects rheumatoid arthritis and cancer development [74]. JAK/STAT regulates CCR5 expression by altering gene transcription in response to cytokine signaling [6]. Cytokines, such as IFN-γ, GFs, and interleukins, activated the JAK/STAT pathway and then up-regulated CCR5 expression [64,75-77]. Gavegnano et al. and our previous work showed that JAK/STAT regulates CCR5 gene expression at both the RNA and cell surface level in human primary T cells [6,78]. Regarding this signaling pathway, there are no anti-STAT compounds, but a total of ten anti-JAK small molecules that are now approved for dermatologic, rheumatic, and gastrointestinal diseases by the FDA in the United States. The European Medicines Agency (EMA) has approved fewer JAK inhibitors (JAKi) for treating a variety of diseases, including rheumatoid arthritis, atopic dermatitis, and ulcerative colitis. It is probable that additional medications will be developed in the future that inhibit JAKs for other illnesses.

The mitogen-activated protein kinase/extracellular signal-regulated kinase (MAPK/ERK) pathway transfers, through a series of steps, the cell surface signal to the nucleus to activate transcription. GFs, such as epidermal GF, TGF, vascular endothelial GF, and various cytokines, activate the MAPK/ERK pathway through receptor tyrosine kinases. This involves a cascade of phosphorylation events, including the activation of Ras, Raf, MEK, and ERK [79]. Activated ERK translocates to the nucleus and phosphorylates transcription factors AP-1 (Fos/Jun) [80] and STAT [65], which then bind related elements in the promoter region of CCR5 and enhance CCR5 transcription [81,82]. However, just as no research has confirmed the existence of STAT binding sites near the CCR5 promoter, Makuta et al. did not find AP-1 binding elements in the CCR5 promoter region using a firefly luciferase reporter [65]. Its exact mechanism, therefore, needs to be further studied.

The phosphoinositide 3-kinase/Ak strain transforming (PI3K/AKT) pathway is also involved in the regulation of CCR5 gene expression. In 2024, it was demonstrated that the binding of CCL5 ligand to CCR5 on prostate cancer cells activates the AKT signaling pathway, resulting in the up-regulation of AR and PD-L1 [15]. GFs and cytokines activate the PI3K/AKT pathway and produce phosphatidylinositol (3,4,5)-trisphosphate, or PIP3. Yang et al. reported that HIV infection of cells did not induce JAK phosphorylation but stimulated the phosphoinositide-dependent kinase-1 and the serine-threonine protein kinase AKT activation, both of which are downstream effectors of PI3K, which recruits and activates AKT, and then further influences the activity of transcription factors, such as NF-κB and AP-1, indirectly affecting CCR5 transcription [83,84]. Additionally, AKT can affect histone modification and influence gene expression [85].

The TGF-β/Suppressor of Mothers against Decapentaplegic (SMAD) signaling pathway is also related to CCR5 gene expression. Generally, TGF-β activates its receptor, leading to SMAD2/3 phosphorylation, and then forms complexes with SMAD4 and translocates to the nucleus, interacting with SMAD binding elements in promoter regions of target genes [86,87]. Last year, it was reported that TGF-β up-regulates CCR5 expression in CD4+ T cells and promotes HIV infection and CD4+ T cell differentiation [88]. However, the mechanism by which this signaling pathway regulates CCR5 expression is not well understood.

Cross-talk between these pathways ensures that the expression of CCR5 is finely regulated in response to diverse extracellular signals. The combined activation of NF-κB and AP-1 may have a synergistic effect on CCR5 transcription regulation, causing a robust inflammatory response. In addition, some pathways can negatively regulate CCR5 expression, such as IL-12 and IL-10 induced pathways, and that results in the recruitment of transcriptional repressors and down-regulation of CCR5 expression [89,90]. Although the mechanisms by which these signaling pathways regulate CCR5 expression are not well understood, drug development can still be pursued based on these pathways.

In summary, understanding and elucidating CCR5 transcriptional regulation mechanisms are central to unraveling certain disease processes and therapeutics, including that of R5-tropic HIV infection of individuals. At present, FDA-approved JAKi that affect CCR5 expression are maraviroc, baricitinib, ruxolitinib, and tofacitinib, at least in our hands, and the precise mechanisms of action deserve further study.

**Figure 3:**
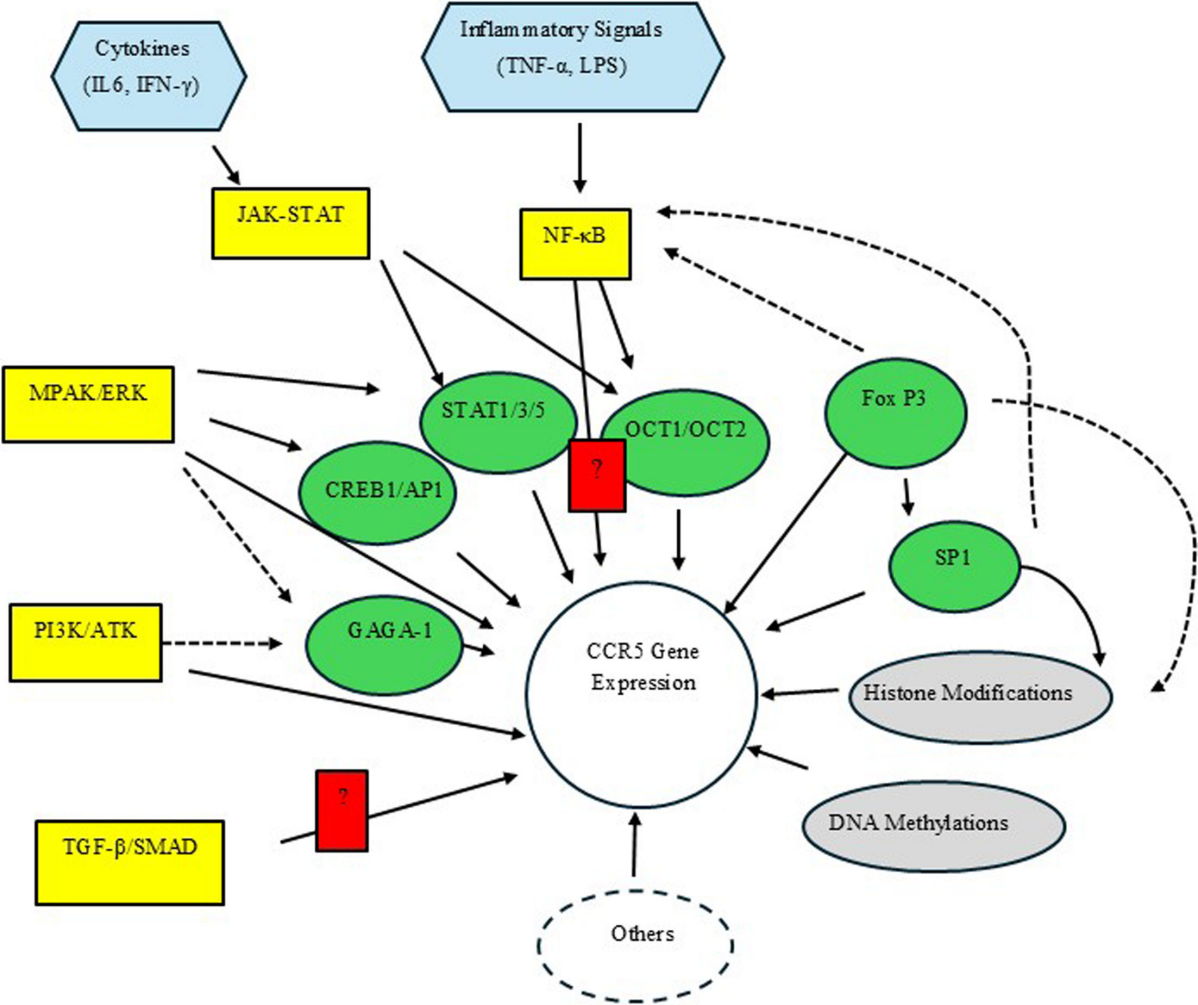
Complex transcription network regulating CCR5 gene expression. Solid lines are confirmed pathways; dashed lines indicate potential regulatory pathways. Grey color shows the genetic and epigenetic regulation, green are transcription factors, yellow are related signaling pathways, and light blue are substrates that activate related signaling pathways. Question marks in red indicate that the precise regulatory pathway is not known. Additional potential regulators of CCR5 gene expression may be found in the future (Others). MPAK/ERK, mitogen-activated protein kinase/extracellular signal-regulated kinase; PI3K/ATK, phosphoinositide 3-kinase/Ak strain transforming; TGF-β/SMAD, transforming growth factor-β/Suppressor of Mothers against Decapentaplegic; JAK, Janus kinase. SP1, specificty protein 1.

To be certain, CCR5 transcriptional regulation is highly complex, and the controlling network likely includes epigenetics, transcription factors, and related signaling pathways (Figure 3). If that is mechanistically correct, further investigation of small molecules or medications that markedly inhibit or reduce CCR5 expression should be pursued if we wish to cure more individuals of R5-tropic HIV. Some of the regulatory mechanisms involved in controlling CCR5 gene expression are not entirely evident, and additional studies are needed.

**Perspectives**

- C-C chemokine receptor type 5 (CCR5) plays a central role in several types of human illnesses, such as human immunodeficiency virus, cancer, graft‐versus‐host disease, and rheumatoid arthritis. Research on CCR5 regulation is critically important to understand disease development and formulate therapeutic strategies. Continued study of CCR5 gene regulation pathways is required.
- Current research has demonstrated that epigenetic modifications, transcription factors, and signaling pathways are involved in CCR5 regulation. Small molecules or medications that target CCR5 epigenetic modifications and signaling pathways should be more fully investigated to affect its gene expression.
- To better define CCR5 gene regulation, although some initial experiments can be performed in immortalized cell lines, more physiological and meaningful results would be obtained in primary human immune cells that normally express CCR5, such as CD4+ T cells and monocyte-derived macrophages. Further investigation of CCR5 regulation by the transforming growth factor-β/Suppressor of Mothers against Decapentaplegic and NF-κB signaling pathways would be very informative. Other transcription factors, enhancers, and suppressors that regulate CCR5 gene expression may be found using modern methodologies, such as ChIP-Seq, RNA-Seq, CRISPR/Cas9 KO, DNase I hypersensitivity studies, and artificial intelligence.

## Open Access

Open access for this article was enabled by the participation of Yale University in an all-inclusive Read & Publish agreement with Portland Press and the Biochemical Society.

## Abbreviations

AIDS: acquired immunodeficiency syndrome
BPD: bronchopulmonary dysplasia
CCR5: C-C chemokine receptor type 5
CRE: cAMP-responsive element
CREB-1: cAMP-responsive element binding protein 1
DA: dopamine
DAC: decitabine
FDA: Federal Drug Agency
GFs: growth factors
GVHD: graft‐versus‐host disease
HIV-1: human immunodeficiency virus type 1
IMs: interstitial macrophages
JAK: Janus kinase
JAKi: Janus kinase inhibitors
MAPK/ERK: mitogen-activated protein kinase/extracellular signal-regulated kinase
MIP: macrophage inflammatory protein
Meth: methamphetamine
NSCLC: non-small cell lung cancer
PI3K/AKT: phosphoinositide 3-kinase/Ak strain transforming
PLWH: people living with HIV
Runx3: runt-related transcription factor 3
SMAD: Suppressor of Mothers against Decapentaplegic
SNPs: single nucleotide polymorphisms
TGF-β: transforming growth factor-β
allo-HSCT: allogeneic hematopoietic stem cell transplantation
cBAF: canonical BAF
lncRNA: long noncoding RNA
m6A: N6-methyladenosine
mRNA: messenger RNA
miRNAs: microRNAs
